# A New Type Fuzzy Module over Fuzzy Rings

**DOI:** 10.1155/2014/730932

**Published:** 2014-03-05

**Authors:** Ece Yetkin, Necati Olgun

**Affiliations:** ^1^Department of Mathematics, Faculty of Sciences and Arts, Marmara University, İstanbul, Turkey; ^2^Department of Mathematics, Faculty of Sciences and Arts, Gaziantep University, Gaziantep, Turkey

## Abstract

A new kind of fuzzy module over a fuzzy ring is introduced by generalizing Yuan and Lee's definition of the fuzzy group and Aktaş and Çağman's definition of fuzzy ring. The concepts of fuzzy submodule, and fuzzy module homomorphism are studied and some of their basic properties are presented analogous of ordinary module theory.

## 1. Introduction

The concept of fuzzy subgroup of a group was first introduced by Rosenfeld [1] in 1971. Since then the theory of fuzzy algebra has been studied by many researchers [2–6]. In the definition of fuzzy subgroups, two types of fuzzy structures are observed in general. In the first type, the subset of a group *G* is fuzzy and the binary operation on *G* is nonfuzzy in the classical sense as Rosenfeld's definition [1]. In the second one, the set is nonfuzzy or classical and the binary operation is fuzzy in fuzzy sense as Yuan and Lee's [7] definition. By the use of Yuan and Lee's definition of fuzzy group based on fuzzy binary operation, Aktaş and Çağman [8] defined a new kind of fuzzy ring.

In this study, we introduce a new kind of fuzzy module by using Yuan and Lee's definition of the fuzzy group and Aktaş and Çağman's definition of fuzzy ring.

The fundamental properties of fuzzy groups and fuzzy rings are presented in Section 2. The concept of fuzzy module is introduced in Section 3. Finally, in Section 4, the concept of fuzzy submodule and fuzzy module homomorphism is presented and a fundamental homomorphism theorem of fuzzy module is obtained.

## 2. Preliminaries

In this section we will formulate the preliminary definitions and results that are required in this paper. Let *θ* ∈ [0,1). Malik and Mordeson [4] gave the following definition.

**Definition 1 (see [4])**

Let *R* and *S* be nonempty sets and let *f* be a fuzzy subset of *R* × *S*; then *f* is called a fuzzy function *R* into *S* if

1. ∀*x* ∈ *R*, ∃*y* ∈ *S* such that *f*(*x*, *y*) > *θ*;
2. ∀*x* ∈ *R*, for all  *y*_1_, *y*_2_ ∈ *S*, *f*(*x*, *y*_1_) > *θ* and *f*(*x*, *y*_2_) > *θ* imply *y*_1_ = *y*_2_.

By the use of Definition 1, Yuan and Lee [7] gave the following definition.

**Definition 2 (see [7])**

Let *G* be a nonempty set and let *R* be a fuzzy subset of *G* × *G* × *G*. *R* is called a fuzzy binary operation on *G* if

1. ∀*a*, *b* ∈ *G*, ∃*c* ∈ *G* such that *R*(*a*, *b*, *c*) > *θ*;
2. ∀*a*, *b*, *c*_1_, *c*_2_ ∈ *G*, *R*(*a*, *b*, *c*_1_) > *θ* and *R*(*a*, *b*, *c*_2_) > *θ* imply *c*_1_ = *c*_2_.

Let *R* be a fuzzy binary operation on *G*; then we have a mapping

(1) $$\begin{array}{c} R:F\left(G\right)\times F\left(G\right)⟶F\left(G\right),\\ \left(A,B\right)⟼R\left(A,B\right), \end{array}$$

where *F*(*G*) = {*A* | *A* : *G* → [0,1]is  a  mapping} and

(2) $$\begin{array}{c} R\left(A,B\right)\left(c\right)=\underset{a,b\in G}{⋁}\left(A\left(a\right)\wedge B\left(b\right)\wedge R\left(a,b,c\right)\right). \end{array}$$

Let *A* = {*a*} and *B* = {*b*} and let *R*(*A*, *B*) be denoted as *a*∘*b*; then

(3) $$\begin{array}{c} \left(a\circ b\right)\left(c\right)=R\left(a,b,c\right),\;\forall c\in G,\\ \left(\left(a\circ b\right)\circ c\right)\left(z\right)=\underset{d\in G}{⋁}\left(R\left(a,b,d\right)\wedge R\left(d,c,z\right)\right),\\ \left(a\circ \left(b\circ c\right)\right)\left(z\right)=\underset{d\in G}{⋁}\left(R\left(b,c,d\right)\wedge R\left(a,d,z\right)\right). \end{array}$$

Using the notations in (3), we have the following.

**Definition 3 (see [7])**

Let *G* be a nonempty set and let *R* be a fuzzy binary operation on *G*.  (*G*, *R*) is called a fuzzy group if the following conditions are true:

(G1) ∀*a*, *b*, *c*, *z*_1_, *z*_2_ ∈ *G*, ((*a*∘*b*)∘*c*)(*z*_1_) > *θ* and (*a*∘(*b*∘*c*))(*z*_2_) > *θ* imply *z*_1_ = *z*_2_;
(G2) ∃*e*_*o*_ ∈ *G* such that (*e*_*o*_∘*a*)(*a*) > *θ* and (*a*  ∘  *e*_*o*_)(*a*) > *θ* for any *a* ∈ *G* (*e*_*o*_ is called an identity element of *G*);
(G3) ∀*a* ∈ *G*, ∃*b* ∈ *G* such that (*a*∘*b*)(*e*_*o*_) > *θ* and (*b*∘*a*)(*e*_*o*_) > *θ* (*b* is called an inverse element of *a* and denoted by *a* ^−1^).

**Proposition 4 (see [7])**

Consider ((*a*∘*b*)∘*c*)(*d*) > *θ*⇔(*a*∘(*b*∘*c*))(*d*) > *θ*.

**Proposition 5 (see [7])**

*H* is a fuzzy subgroup of *G* if and only if

1. ∀*a*, *b* ∈ *H*, ∀*c* ∈ *G*, (*a*∘*b*)(*c*) > *θ* *implies* *c* ∈ *H*;
2. *a* ∈ *H* *implies* *a* ^−1^ ∈ *H*.

**Definition 6 (see [7])**

Let *H* be a fuzzy subgroup of *G*. Let

(4) $$\begin{array}{c} \left(aH\right)\left(z\right)=\underset{x\in H}{⋁}R\left(a,x,z\right);\;\;\left(Ha\right)\left(z\right)=\underset{x\in H}{⋁}R\left(x,a,z\right), \end{array}$$

and *aH*  (*Ha*) is called a left (right) coset of *H*.

**Definition 7 (see [7])**

Let *H* be a fuzzy subgroup of *G*:

(5) $$\begin{array}{c} \forall a,b\in G,\;\;\forall h\in H,\;\left(a\circ \left(h\circ a^{-1}\right)\right)\left(b\right)>\theta ⟹b\in H, \end{array}$$

and then *H* is called a normal fuzzy subgroup of *G*.

**Definition 8 (see [8])**

Let (*G*, *R*) be a fuzzy subgroup. If

(6) $$\begin{array}{c} \left(a\circ b\right)\left(c\right)>\theta ⟺\left(b\circ a\right)\left(c\right)>\theta ,\;\forall a,b,c\in G, \end{array}$$

then (*G*, *R*) is called abelian fuzzy group.

**Theorem 9 (see [7])**

Let [*aH*] = {*a*′*H* | *a*′*H* ~ *aH*}, $\overset{-}{a}=\{a^{\prime }|a^{\prime }\in G$  and  *a*′*H* ~ *aH*}, *G*/*H* = {[*aH*] | *a* ∈ *G*}, and

(7) R−:GH×GH×GH⟶[0,1],([aH],[bH],[cH])⟼R−([aH],[bH],[cH])=⋁(a′,b′,c′)∈a−×b−×c−R(a′,b′,c′),

and then $\overset{-}{R}$ is a fuzzy binary relation on *G*/*H*.

**Theorem 10 (see [7])**

$\left(G/H,\overset{-}{R}\right)$ is a fuzzy group.

**Definition 11 (see [7])**

Let (*G*_1_, *R*_1_) and (*G*_2_, *R*_2_) be two fuzzy groups and let *f* : *G*_1_ → *G*_2_ be a mapping. If

(8) $$\begin{array}{c} R_{1}\left(a,b,c\right)>\theta ⟹R_{2}\left(f\left(a\right),f\left(b\right),f\left(c\right)\right)>\theta , \end{array}$$

then *f* is called a fuzzy (group) homomorphism. If *f* is 1-1, it is called a fuzzy monomorphism. If *f* is onto, it is called a fuzzy epimorphism. If *f* is both 1-1 and onto, it is called a fuzzy isomorphism.

Let *G* be a fuzzy binary operation on *R*. Then we have a mapping

(9) $$\begin{array}{c} G:F\left(R\right)\times F\left(R\right)⟶F\left(R\right),\\ \left(A,B\right)⟼G\left(A,B\right), \end{array}$$

where *F*(*R*) = {*A* | *A* : *R* → [0,1]  is  a  mapping} and

(10) $$\begin{array}{c} G\left(A,B\right)\left(c\right)=\underset{a,b\in R}{⋁}\left(A\left(a\right)\wedge B\left(b\right)\wedge G\left(a,b,c\right)\right). \end{array}$$

Let *A* = {*a*} and *B* = {*b*} and let *G*(*A*, *B*) and *H*(*A*, *B*) be denoted as *a*∘*b* and *a*∗*b*, respectively. Then

(11) (a∘b)(c)=G(a,b,c), ∀c∈R,(a∗b)(c)=H(a,b,c), ∀c∈R,((a∘b)∘c)(z)=⋁d∈G(G(a,b,d)∧G(d,c,z)),(a∘(b∘c))(z)=⋁d∈G(G(b,c,d)∧G(a,d,z)),(a∗(b∘c))(z)=⋁d∈G(G(b,c,d)∧H(a,d,z)),((a∗b)∘(a∗c))(z)  =⋁d∈G(H(a,b,d)∧H(a,c,e)∧G(d,e,z)).

Using the notations of (11), we have the following.

**Definition 12 (see [8])**

Let *R* be a nonempty set and let *G* and *H* be two fuzzy binary operations on *R*. Then (*R*, *G*, *H*) is called fuzzy ring if the following conditions hold:

(R1) (*R*, *G*) is an abelian fuzzy group;
(R2) ∀*a*, *b*, *c*, *z*_1_, *z*_2_ ∈ *R*, ((*a*∗*b*)∗*c*)(*z*_1_) > *θ* and (*a*∗(*b*∗*c*))(*z*_2_) > *θ* imply *z*_1_ = *z*_2_;
(R3) ∀*a*, *b*, *c*, *z*_1_, *z*_2_ ∈ *R*, ((*a*∘*b*)∗*c*)(*z*_1_) > *θ* and ((*a*∗*c*)∘(*b*∗*c*))(*z*_2_) > *θ* imply *z*_1_ = *z*_2_; (*a*∗(*b*∘*c*))(*z*_1_) > *θ* and ((*a*∗*b*)∘(*a*∗*c*))(*z*_2_) > *θ* imply *z*_1_ = *z*_2_.

**Definition 13 (see [8])**

Let (*R*, *G*, *H*) be a fuzzy ring.

1. If (*a*∗*b*)(*u*) > *θ*⇔(*b*∗*a*)(*u*) > *θ*, then (*R*, *G*, *H*) is said to be a commutative fuzzy ring.
2. If ∃*e*_∗_ ∈ *R* such that (*a*∗*e*_∗_)(*a*) > *θ* and (*e*_∗_∗*a*)(*a*) > *θ* for every *a* ∈ *R*, then (*R*, *G*, *H*) is said to be fuzzy ring with identity.
3. Let (*R*, *G*, *H*) be a fuzzy ring with identity. If (*a*∗*b*)(*e*_∗_) > *θ* and (*b*∗*a*)(*e*_∗_) > *θ*, ∀*a* ∈ *R*, ∃*b* ∈ *R*, then *b* is said to be an inverse element of *a* and is denoted by *a*_∗_^−1^.

**Proposition 14 (see [8])**

Let (*R*, *G*, *H*) be a fuzzy ring and let *S* be a nonempty subset of *R*. Then (*S*, *G*, *H*) is a fuzzy subring of *R* if and only if

1. (*a*∘*b*)(*c*) > *θ* *implies* *c* ∈ *S* *and*  (*a*∗*b*)(*c*) > *θ* *implies* *c* ∈ *S* *for all* *a*, *b* ∈ *S*, *c* ∈ *R*;
2. *a* ∈ *S* *implies* *a* ^−1^ ∈ *S* *for all* *a* ∈ *S*.

**Definition 15 (see [8])**

A nonempty subset *I* of a fuzzy ring (*R*, *G*, *H*) is called a fuzzy ideal of *R* if the following conditions are satisfied.

1. ∀*x*, *y* ∈ *I*, (*x*∘*y*)(*z*) > *θ*⇒*z* ∈ *I* for all *z* ∈ *R*;
2. ∀*x* ∈ *I*, *x* ^−1^ ∈ *I*;
3. For all *s* ∈ *I*, for all *r* ∈ *R*, (*r*∗*s*)(*x*) > *θ*⇒*x* ∈ *I* and (*s*∗*r*)(*y*) > *θ*⇒*y* ∈ *I*, *x*, *y* ∈ *R*.

**Definition 16 (see [8])**

Let *I* be a fuzzy ideal of fuzzy ring *R* and let *Ω* = {*a*∘*I* | *a* ∈ *R*}. One defines a relation over *Ω*:

(12) $$\begin{array}{c} a_{1}\circ I∼a_{2}\circ I⟺\exists u\in I\;\;\text{such}\;\;\text{that}\;\;G\left(a_{1}^{-1},a_{2},u\right)>\theta . \end{array}$$

## 3. Fuzzy Modules over Fuzzy Rings

Let (*R*, *G*, *H*) be a fuzzy ring and (*M*, *J*) be an abelian fuzzy group and let *p* be fuzzy function *R* × *M* into *M*. Then we have a mapping

(13) $$\begin{array}{c} p:F\left(R\right)\times F\left(M\right)⟶F\left(M\right),\\ \left(A,N\right)⟼p\left(A,N\right),\\ p\left(A,N\right)\left(x\right)=\underset{\left(r,n\right)\in A\times N}{⋁}\left(A\left(r\right)\wedge N\left(n\right)\wedge p\left(r,n,x\right)\right), \end{array}$$

where *F*(*R*) = {*A* | *A* : *R* → [0,1]  is  a  mapping} and *F*(*M*) = {*N* | *N* : *M* → [0,1]is  a  mapping}.

Let *A* = {*r*} and *N* = {*m*}, and let *p*(*A*, *N*) and *J*(*a*, *b*) be denoted as *r*⊙*m* and *a* ⊕ *b*, respectively. Then

(14) (r⊙m)(x)=p(r,m,x), ∀x∈M,(r⊙(m1⊕m2))(x)=⋁m∈M(J(m1,m2,m)∧p(r,m,x)),((r1∘r2)⊙m)(x)=⋁r∈R(G(r1,r2,r)∧p(r,m,x)),((r1∗r2)⊙m)(x)=⋁r∈R(H(r1,r2,r)∧p(r,m,x)),(r1⊙(r2⊙m))(x)=⋁m1∈M(p(r2,m,m1)∧p(r1,m1,x)),((r⊙m1)⊕(r⊙m2))(x)  =⋁x1,x2∈M(p(r,m1,x1)∧p(r,m2,x2)∧J(x1,x2,x)).

Using the notations (14), we have the following.

**Definition 17**

Let (*R*, *G*, *H*) be a fuzzy ring and let (*M*, *J*) be an abelian fuzzy group. *M* is called a (left) fuzzy module over *R* or (left) *R*-fmodule together with a fuzzy function *p* : *R* × *M* → *M* if the following conditions hold. For all *r*, *r*_1_, *r*_2_ ∈ *R* and for all *m*, *m*_1_, *m*_2_ ∈ *M*,

(M1) (*r*⊙(*m*_1_ ⊕ *m*_2_))(*x*) > *θ* and ((*r*⊙*m*_1_)⊕(*r* ⊙ *m*_2_))(*y*) > *θ* imply *x* = *y*;
(M2) ((*r*_1_∘*r*_2_)⊙*m*)(*x*) > *θ* and ((*r*_1_⊙*m*)⊕(*r*_2_⊙*m*))(*y*) > *θ* imply *x* = *y*;
(M3) ((*r*_1_∗*r*_2_)  ⊙  *m*)(*x*) > *θ* and (*r*_1_  ⊙  (*r*_2_  ⊙  *m*))(*y*) > *θ* imply *x* = *y*.

**Proposition 18**

Let (*R*, *G*, *H*) be a fuzzy ring and let (*M*, *J*) be an *R*-fmodule; then for all *r*, *r*_1_, *r*_2_ ∈ *R*, *m*, *m*_1_, *m*_2_ ∈ *M*,

1. (*r* ⊙ (*m*_1_ ⊕ *m*_2_))(*x*) > *θ*⇔((*r*  ⊙  *m*_1_)⊕(*r*  ⊙  *m*_2_))(*x*) > *θ*;
2. ((*r*_1_∘*r*_2_)⊙*m*)(*x*) > *θ*  ⇔  ((*r*_1_⊙*m*)⊕(*r*_2_⊙*m*))(*x*) > *θ*;
3. ((*r*_1_∗*r*_2_)⊙*m*)(*x*) > *θ*  ⇔  (*r*_1_⊙(*r*_2_⊙*m*))(*x*) > *θ*.

**Proof**

(1) Let (*r*  ⊙  (*m*_1_  ⊕  *m*_2_))(*x*) > *θ* and let *x*_1_, *x*_2_, *y* ∈ *M* such that *p*(*r*, *m*_1_, *x*_1_) > *θ*, *p*(*r*, *m*_2_, *x*_2_) > *θ*, and *J*(*x*_1_, *x*_2_, *y*) > *θ*. By

(15) ((r⊙m1)⊕(r⊙m2))(y)  >p(r,m1,x1)∧p(r,m2,x2)∧J(x1,x2,y)>θ

we get *x* = *y* from (M1) and so ((*r*  ⊙  *m*_1_) ⊕ (*r*  ⊙  *m*_2_))(*x*) > *θ*. Similarly by ((*r*⊙*m*_1_)⊕(*r*⊙*m*_2_))(*x*) > *θ* we have (*r*⊙(*m*_1_ ⊕ *m*_2_))(*x*) > *θ*.

It is easy to prove (2) and (3) similar to the proof of (1).

**Proposition 19**

Let (*R*, *G*, *H*) be a fuzzy ring with zero element *e*_*o*_ and (*M*, *J*) be a left *R*-fmodule with identity element *e*_*J*_. Then for all *r* ∈ *R*, *m* ∈ *M*,

1. (*r*⊙*e*_*J*_)(*e*_*J*_) > *θ*;
2. (*e*_*o*_⊙*m*)(*e*_*J*_) > *θ*;
3. (*r*⊙*m*)(*x*) > *θ*⇒(*r*⊙*m* ^−1^)(*x* ^−1^) > *θ*;
4. (*r*⊙*m*)(*x*) > *θ*⇒(*r* ^−1^⊙*m*)(*x* ^−1^) > *θ*.

**Proof**

(1) Let *x* ∈ *M* such that (*r*⊙*e*_*J*_)(*x*) > *θ*. Then  

(16) $$\begin{array}{c} \left(r⊙\left(e_{J}⊕e_{J}\right)\right)\left(x\right)>J\left(e_{J},e_{J},e_{J}\right)\wedge p\left(r,e_{J},x\right)>\theta . \end{array}$$

It follows that ((*r*  ⊙  *e*_*J*_) ⊕ (*r*  ⊙  *e*_*J*_))(*x*) > *θ* from Proposition 18. Then

(17) ((r⊙eJ)⊕(r⊙eJ))(x)  >p(r,eJ,x)∧p(r,eJ,x)∧J(x,x,x)>θ.

Thus *J*(*x*, *x*, *x*) > *θ* and *x* = *e*_*J*_ from Proposition  2.1 in [7].

(2) Let *x* ∈ *M* such that (*e*_*o*_⊙*m*)(*x*) > *θ*. Then

(18) $$\begin{array}{c} \left(\left(e_{o}\circ e_{o}\right)⊙m\right)\left(x\right)>G\left(e_{o},e_{o},e_{o}\right)\wedge p\left(e_{o},m,x\right)>\theta . \end{array}$$

It follows that ((*e*_*o*_⊙*m*)⊕(*e*_*o*_⊙*m*))(*x*) > *θ* from Proposition 18. Then

(19) ((eo⊙m)⊕(eo⊙m))(x)  >p(eo,m,x)∧p(eo,m,x)∧J(x,x,x)>θ.

Thus similar to (1), *J*(*x*, *x*, *x*) > *θ* and so *x* = *e*_*J*_.

(3) Let *p*(*r*, *m*, *x*) > *θ* and let *y* ∈ *M* such that *p*(*r*, *m* ^−1^, *y*) > *θ*. Since

(20) $$\begin{array}{c} \left(r⊙\left(m⊕m^{-1}\right)\right)\left(e_{J}\right)>J\left(m,m^{-1},e_{J}\right)\wedge p\left(r,e_{J},e_{J}\right)>\theta , \end{array}$$

by Proposition 18 we have ((*r* ⊙ *m*) ⊕ (*r* ⊙ *m* ^−1^))(*e*_*J*_) > *θ*. Hence

(21) ((r⊙m)⊕(r⊙m−1))(eJ)  >p(r,m,x)∧p(r,m−1,y)∧J(x,y,eJ)>θ.

Therefore *J*(*x*, *y*, *e*_*J*_) > *θ* and consequently *y* = *x* ^−1^.

(4) It is obtained similar to (3).

**Proposition 20**

If (*R*, *G*, *H*) is a fuzzy ring and *K* is any fuzzy subring of *R*, then *R* is a left *K*-fmodule.

**Proof**

Let (*R*, *G*, *H*) be a fuzzy ring and let (*K*, *G*, *H*) be a fuzzy subring of *R*. Consider the mapping

(22) $$\begin{array}{c} p:K\times R⟶R, \end{array}$$

defined by *p*(*k*, *r*) = *H*(*k*, *r*). It is obviously a fuzzy function which satisfies the conditions in Definition 17. Moreover observe that (*R*, *G*) is necessarily an abelian fuzzy group. Consequently *R* is a left *K*-fmodule.

## 4. Fuzzy Submodule and Fuzzy Module Homomorphism

**Definition 21**

Let (*R*, *G*, *H*) be a fuzzy ring, (*M*, *J*) an *R*-fmodule, and *N* a nonempty subset of *M*. If (*N*, *J*) is an *R*-fmodule, *N* is called a fuzzy submodule of *M*.

**Proposition 22**

Let (*R*, *G*, *H*) be a fuzzy ring, (*M*, *J*) an *R*-fmodule, and *N* a nonempty subset of *M*. Then *N* is a fuzzy submodule of *M* if and only if

1. (*N*, *J*) *is a fuzzy subgroup of*  (*M*, *J*);
2. *for all* *r* ∈ *R*, *b* ∈ *N*, (*r*⊙*b*)(*c*) > *θ* *implies* *c* ∈ *N*.

**Proposition 23**

If  {*N*_*i*_ | *i* ∈ *I*} is a family of fuzzy submodules of a fuzzy module *M*, then ⋂_*i*∈*I*_ *N*_*i*_ is a fuzzy submodule of *M*.

**Definition 24**

Let *A* and *B* be two fuzzy modules over a fuzzy ring (*R*, *G*, *H*) with a function *p* : *R* × *M* → *M*. A function *f* : *A* → *B* is an *R*-fmodule homomorphism which provided that, for all *a*, *b* ∈ *A* and *r* ∈ *R*,

1. *G*(*a*, *b*, *x*) > *θ* implies *G*(*f*(*a*), *f*(*b*), *f*(*x*)) > *θ*;
2. *p*(*r*, *a*, *x*) > *θ* implies *p*(*r*, *f*(*a*), *f*(*x*)) > *θ*.

Clearly, an *R*-fmodule homomorphism *f* : *A* → *B* is necessarily an abelian fuzzy group homomorphism. Consequently the same terminology is used for fuzzy modules: *f* is an *R*-fmodule monomorphism (resp., epimorphism, isomorphism) if it is injective (resp., surjective, bijective) as fuzzy group homomorphisms.

Let *f* : *A* → *B* be an *R*-fmodule homomorphism. Then the kernel and the image of *f* as fuzzy group homomorphisms are denoted by

(23) Ker⁡ f={a∈A ∣ f(a)=eB},Im⁡ f={b∈B ∣ b=f(a),a∈A},

respectively.

**Theorem 25**

Let (*R*, *G*, *H*) be a fuzzy ring and let *f* : *A* → *B* be an *R*-fmodule homomorphism. Then

1. *f* is an *R*-fmodule monomorphism if and only if Ker⁡ *f* = {*e*_*A*_};
2. *f* : *A* → *B* is an *R*-fmodule isomorphism if and only if there exists a fuzzy module homomorphism *g* : *B* → *A* such that *gf* = *e*_*A*_ and *fg* = *e*_*B*_.

**Proposition 26**

Let *f* : *A* → *B* be an *R*-fmodule homomorphism. Then

1. Ker⁡ *f* *is a fuzzy submodule of* *A*;
2. *Im*⁡ *f* *is a fuzzy submodule of* *B*;
3. *if* *C* *is any fuzzy submodule of* *B*,  *then* *f* ^−1^(*C*) = {*a* ∈ *A* | *f*(*a*) ∈ *C*}  *is a fuzzy submodule of* *A*.

**Proof**

(1) Ker⁡ *f* is a fuzzy subgroup of the abelian fuzzy group *A* from Theorem  26 in [8]. Let *r* ∈ *R* and *a* ∈ Ker⁡  *f* such that *p*(*r*, *a*, *x*) > *θ*. Since *f* is an *R*-fmodule homomorphism, *p*(*r*, *f*(*a*), *f*(*x*)) > *θ*. On the other hand, as *a* ∈ Ker⁡  *f* we have *f*(*a*) = *e*_*B*_. Therefore *p*(*r*, *e*_*B*_, *f*(*x*)) > *θ* and so *f*(*x*) = *e*_*B*_ from Proposition 19. So *x* ∈ Ker⁡ *f* is obtained.

(2) *Im*⁡  *f* is a fuzzy subgroup of the abelian fuzzy group *A* from Theorem  26 in [8]. For any *r* ∈ *R*, *b* ∈ *Im*⁡  *f*, there exists *a* ∈ *A* such that *b* = *f*(*a*). Let *x* ∈ *A* such that *H*(*r*, *a*, *x*) > *θ*. Since *f* is an *R*-fmodule homomorphism, *H*(*r*, *f*(*a*), *f*(*x*)) > *θ* which means *H*(*r*, *b*, *f*(*x*)) > *θ* and so *f*(*x*) ∈ *B*.

(3) *f* ^−1^(*C*) is a fuzzy subgroup of the abelian fuzzy group *A* from Theorem  5.2 in [7]. Let *r* ∈ *R* and *x* ∈ *f* ^−1^(*C*) such that *H*(*r*, *x*, *u*) > *θ*. Since *H*(*r*, *f*(*x*), *f*(*u*)) > *θ* and *f*(*x*) ∈ *C*, we have that *f*(*u*) ∈ *C* and *u* ∈ *f* ^−1^(*C*). This completes the proof.

**Proposition 27**

Let *I* be a left fuzzy ideal of a fuzzy ring (*R*, *G*, *H*), (*A*, *J*) an *R*-fmodule, and *a* ∈ *A*. Consider the set *B* = *I*∗*a* = {*x* ∈ *R* | *H*(*r*, *a*, *x*) > *θ*, *r* ∈ *I*}. Then

1. *B* *is a fuzzy submodule of* *A*;
2. *The map* *φ* : *I* → *B* *given by* *φ*(*r*) = *r*∗*a* *is an* *R-fmodule epimorphism.*

**Proof**

(1) First we show that *B* is a fuzzy subgroup of *A*. Let *x*, *y* ∈ *B*; then there exist *r*_1_, *r*_2_ ∈ *I* such that *H*(*r*_1_, *a*, *x*) > *θ* and *H*(*r*_2_, *a*, *y*) > *θ*. From Proposition 19, *H*(*r*_2_, *a*, *y*) > *θ* implies *H*(*r*_2_^−1^, *a*, *y* ^−1^). Since *I* is a left fuzzy ideal, there exists *r* ∈ *I* such that *G*(*r*_1_, *r*_2_^−1^, *r*) > *θ*.

Let *u* ∈ *B* such that *H*(*r*, *a*, *u*) > *θ*. Then

(24) $$\begin{array}{c} \left(\left(r_{1}\circ r_{2}^{-1}\right)*a\right)\left(u\right)>G\left(r_{1},r_{2}^{-1},r\right)\wedge H\left(r,a,u\right)>\theta . \end{array}$$

On the other hand, since *H*(*r*_2_^−1^, *a*, *y* ^−1^) > *θ* and ((*r*_1_∗*a*) ⊕ (*r*_2_^−1^∗*a*))(*u*) > *θ* from Proposition 18, we obtain

(25) ((r1∗a)⊕(r2−1∗a))(u)  >H(r1,a,x)∧H(r2−1,a,y−1)∧J(x,y−1,u)>θ.

Thus *J*(*x*, *y* ^−1^, *u*) > *θ*, *u* ∈ *B*, which means that (*B*, *J*) is a fuzzy subgroup of (*A*, *J*).

Now consider a mapping *p* : *R* × *B* → *B* defined by *p*(*r*, *b*) = *H*(*r*, *b*). Let (*r*⊙*b*)(*c*) > *θ*, for any *r* ∈ *R*, *b* ∈ *B*. Since *b* ∈ *B*, there exists *r* ∈ *I* such that *H*(*r*, *a*, *b*) > *θ*.

Let *s* ∈ *I* such that *H*(*r*, *r*, *s*) > *θ*. Then

(26) $$\begin{array}{c} \left(r⊙\left(r⊙a\right)\right)\left(c\right)>H\left(r,a,b\right)\wedge H\left(r,b,c\right)>\theta , \end{array}$$

and it follows that ((*r*∗*r*)⊙*a*)(*c*) > *θ*. Thus

(27) $$\begin{array}{c} \left(\left(r*r\right)⊙a\right)\left(c\right)>H\left(r,r,s\right)\wedge H\left(s,a,c\right)>\theta , \end{array}$$

and we have *H*(*s*, *a*, *c*) > *θ*. Since *s* ∈ *I*, *c* ∈ *B* is obtained. Therefore *B* is a fuzzy submodule of *A*.

(2) Let *r*, *r*_1_, *r*_2_ ∈ *I* such that *G*(*r*_1_, *r*_2_, *r*) > *θ* and let *x*, *x*_1_, *x*_2_ ∈ *B* such that *φ*(*r*) = *x*, *φ*(*r*_1_) = *x*_1_, and *φ*(*r*_2_) = *x*_2_. So, *H*(*r*, *a*, *x*) > *θ*,  *H*(*r*_1_, *a*, *x*_1_) > *θ*, and *H*(*r*_2_, *a*, *x*_2_) > *θ*. Since

(28) $$\begin{array}{c} \left(\left(r_{1}\circ r_{2}\right)*a\right)\left(x\right)>G\left(r_{1},r_{2},r\right)\wedge H\left(r,a,x\right)>\theta , \end{array}$$

we have

(29) ((r1∗a)⊕(r2∗a))(x)  >H(r1,a,x1)∧H(r2,a,x2)∧J(x1,x2,x)>θ.

It follows that *J*(*x*_1_, *x*_2_, *x*) > *θ* which means *J*(*φ*(*r*_1_), *φ*(*r*_2_), *φ*(*r*)) > *θ*.

Finally we show that if *r* ∈ *R*, *k* ∈ *I* such that (*r*  ⊙  *k*)(*s*) > *θ*, then (*r*⊙*φ*(*k*))(*φ*(*s*)) > *θ*. For this purpose, let *φ*(*k*) = *x* ∈ *B* and *φ*(*s*) = *y* ∈ *B*; then *H*(*k*, *a*, *x*) > *θ* and *H*(*s*, *a*, *y*) > *θ*. Since

(30) $$\begin{array}{c} \left(\left(r*k\right)*a\right)\left(y\right)>H\left(r,k,s\right)\wedge H\left(s,a,y\right)>\theta , \end{array}$$

we have

(31) $$\begin{array}{c} \left(r*\left(k*a\right)\right)\left(y\right)>H\left(k,a,x\right)\wedge H\left(r,x,y\right)>\theta , \end{array}$$

and it follows that *H*(*r*, *x*, *y*) > *θ*, and consequently we obtain (*r*  ⊙  *φ*(*k*))(*φ*(*s*)) > *θ*. *φ* : *I* → *B* is obviously surjective and *φ* is an *R*-fmodule epimorphism.

**Proposition 28**

Let (*R*, *G*, *H*) be a fuzzy ring and *B* a fuzzy submodule of an *R*-fmodule (*A*, *J*) and let *a*_1_ ⊕ *B* ~ *a*_2_ ⊕ *B*, *a* ⊕ *B* ~ *a*′ ⊕ *B*, and *c* ⊕ *B* ~ *c*′ ⊕ *B*. Consider a mapping defined by

(32) p¯:R×AB×AB⟶[0,1],p¯(r,[a⊕B],[c⊕B])  =(r⊙[a⊕B])([c⊕B])=⋁(a′,c′)∈a−×c−p(r,a′,c′).

If *p*(*r*, *a*_1_, *x*) > *θ* and *p*(*r*, *a*_2_, *y*) > *θ*, then *x* ⊕ *B* ~ *y* ⊕ *B*.

**Proof**

Let *p*(*r*, *a*_1_, *x*) > *θ* and *p*(*r*, *a*_2_, *y*) > *θ*. Since *a*_1_ ⊕ *B* ~ *a*_2_ ⊕ *B* and by Definition 16, there exists *u* ∈ *B* such that *J*(*a*_1_^−1^, *a*_2_, *u*) > *θ*. Let *a* ∈ *A* and *v* ∈ *B* such that *J*(*a*_1_, *a*_2_, *a*) > *θ* and *p*(*r*, *u*, *v*) > *θ*. As *B* is an *R*-fmodule, *v* ∈ *B*. Then

(33) $$\begin{array}{c} \left(r⊙\left(a_{1}^{-1}⊕a_{2}\right)\right)\left(v\right)>J\left(a_{1}^{-1},a_{2},u\right)\wedge p\left(r,u,v\right)>\theta , \end{array}$$

Let ((*r*  ⊙  *a*_1_^−1^)  ⊕  (*r*  ⊙  *a*_2_))(*w*) > *θ*. By Proposition 19, *p*(*r*, *a*_1_, *x*) > *θ* implies *p*(*r*, *a*_1_^−1^, *x* ^−1^) > *θ*. Hence

(34) ((r⊙a1−1)⊕(r⊙a2))(w)  >p(r,a1−1,x−1)∧p(r,a2,y)∧J(x−1,y,w)>θ.

Thus *w* = *v*, so *J*(*x* ^−1^, *y*, *v*) > *θ* and consequently *x* ⊕ *B* ~ *y* ⊕ *B*.

Then we have the following result.

**Theorem 29**

Let (*R*, *G*, *H*) be a fuzzy ring and *B* a fuzzy submodule of an *R*-fmodule (*A*, *J*). Then *A*/*B* is an *R*-fmodule with the mapping $\bar{p}$ defined in Proposition 28.

**Proof**

Since (*A*, *J*) is an abelian fuzzy group, (*B*, *J*) is necessarily a normal fuzzy subgroup of *A*. Hence the abelian factor fuzzy group *A*/*B* is well-defined. Since *A* is an *R*-fmodule, for all *r* ∈ *R*, *a*_1_ ∈ *A*, there exists *a*_2_ ∈ *A* such that *p*(*r*, *a*_1_, *a*_2_) > *θ*. Then $\bar{p}(r[a_{1}⊕B],[a_{2}⊕B])>p(r,a_{1},a_{2})>\theta$.

Let (*r*⊙[*a* ⊕ *B*])([*x* ⊕ *B*]) > *θ* and (*r*⊙[*a* ⊕ *B*])([*y* ⊕ *B*]) > *θ*. Then there exist $a_{1},a_{2}\in \bar{a}$, $x^{\prime }\in \bar{x}$, and $y^{\prime }\in \bar{y}$ such that *p*(*r*, *a*_1_, *x*′) > *θ* and *p*(*r*, *a*_2_, *y*′) > *θ*. Since *a*_1_ ⊕ *B* ~ *a*_2_ ⊕ *B*, we have *x*′ ⊕ *B* ~ *y*′ ⊕ *B* from Proposition 28 and consequently [*x* ⊕ *B*] = [*y* ⊕ *B*].

(1) Let (*r*⊙([*a*_1_ ⊕ *B*]⊕[*a*_2_ ⊕ *B*]))([*x* ⊕ *B*]) > *θ* and ((*r*⊙[*a*_1_ ⊕ *B*])⊕(*r*⊙[*a*_2_ ⊕ *B*]))([*y* ⊕ *B*]) > *θ* and let *a* ∈ *A* such that *J*(*a*_1_, *a*_2_, *a*) > *θ*. Then there exist  $a_{1}^{\prime }\in \bar{a_{1}}$, $a_{2}^{\prime }\in \bar{a_{2}}$, *x*′, *x*_1_′, *x*_2_′ ∈ *A*, $y^{\prime }\in \bar{y}$, and *b*_1_, *b*_2_ ∈ *B* such that

(35) $$\begin{array}{c} J\left(a_{1},a_{2},a\right)\wedge p\left(r,a^{\prime },x^{\prime }\right)>\theta ,\\ p\left(r,a_{1}^{\prime },x_{1}^{\prime }\right)\wedge p\left(r,a_{2}^{\prime },x_{2}^{\prime }\right)\wedge J\left(x_{1}^{\prime },x_{2}^{\prime },y^{\prime }\right)>\theta ,\\ J\left(a_{1}^{\prime },b_{1},a_{1}\right)>\theta ,\;\;J\left(a_{2}^{\prime },b_{2},a_{2}\right)>\theta . \end{array}$$

Let *w* ∈ *A* such that *J*(*a*_1_′, *a*_2_′, *w*) > *θ*. Then by *J*(*a*_1_′, *b*_1_, *a*_1_) > *θ*, *J*(*a*_2_′, *b*_2_, *a*_2_) > *θ*, *J*(*a*_1_, *a*_2_, *a*) > *θ*, and the proof of Theorem  4.2 in [7], we get *b* ∈ *B* such that *J*(*w*, *b*, *a*) > *θ*. Then *J*(*a*, *b* ^−1^, *w*) > *θ*. Since

(36) ((r⊙a1′)⊕(r⊙a2′))(y′)  >p(r,a1′,x1′)∧p(r,a2′,x2′)∧J(x1′,x2′,y′)>θ

and *A* is an *R*-fmodule, we have (*r*⊙(*a*_1_′ ⊕ *a*_2_′))(*y*′) > *θ*. So

(37) $$\begin{array}{c} \left(r⊙\left(a_{1}^{\prime }⊕a_{2}^{\prime }\right)\right)\left(y^{\prime }\right)>J\left(a_{1}^{\prime },a_{2}^{\prime },w\right)\wedge p\left(r,w,y^{\prime }\right)>\theta . \end{array}$$

Hence *p*(*r*, *w*, *y*′) > *θ*. Then

(38) $$\begin{array}{c} \left(r⊙\left(a⊕b^{-1}\right)\right)\left(y^{\prime }\right)>J\left(a,b^{-1},w\right)\wedge p\left(r,w,y^{\prime }\right). \end{array}$$

Let *x*_3_ ∈ *A*, *b*_3_ ∈ *B* such that *p*(*r*, *a*, *x*_3_) > *θ* and *p*(*r*, *b* ^−1^, *b*_3_) > *θ*. Then

(39) ((r⊙a)⊙(r⊙b−1))(y′)  >p(r,a,x3)∧p(r,b−1,b3)∧J(x3,b3,y′)>θ.

It follows that *J*(*x*_3_, *b*_3_, *y*′) > *θ* and so *x*_3_ ⊕ *B* ~ *y*′ ⊕ *B*. Therefore we obtain *x*′ ⊕ *B* ~ *y*′ ⊕ *B* and consequently [*x*  ⊕  *B*] = [*y* ⊕ *B*].

(2) Let ((*r*_1_∘*r*_2_)⊙[*a* ⊕ *B*])([*x* ⊕ *B*]) > *θ* and ((*r*_1_⊙[*a* ⊕ *B*]) ⊕ (*r*_2_⊙[*a* ⊕ *B*]))([*y* ⊕ *B*]) > *θ* and let *r* ∈ *R* such that *G*(*r*_1_, *r*_2_, *r*) > *θ*. Then we have $a_{1},a_{2},a_{3}\in \bar{a}$, $x_{1}\in \bar{x}$, $y_{1}\in \bar{y}$, *x*_2_, *x*_3_ ∈ *A*, and *b*_1_, *b*_2_ ∈ *B* such that

(40) $$\begin{array}{c} G\left(r_{1},r_{2},r\right)\wedge p\left(r,a_{1},x_{1}\right)>\theta ,\\ p\left(r_{1},a_{2},x_{2}\right)\wedge p\left(r_{2},a_{3},x_{3}\right)\wedge J\left(x_{2},x_{3},y_{1}\right)>\theta ,\\ J\left(a_{1},b_{1},a_{2}\right)>\theta ,\;J\left(a_{1},b_{2},a_{3}\right)>\theta . \end{array}$$

Then, by

(41) $$\begin{array}{c} \left(r_{1}⊙\left(a_{1}⊕b_{1}\right)\right)\left(x_{2}\right)>J\left(a_{1},b_{1},a_{2}\right)\wedge p\left(r_{1},a_{2},x_{2}\right)>\theta , \end{array}$$

we have ((*r*_1_⊙*a*_1_) ⊕ (*r*_1_⊙*b*_1_))(*x*_2_) > *θ*. So there exist *u*_1_ ∈ *A*, *v*_1_ ∈ *B* such that

(42) $$\begin{array}{c} p\left(r_{1},a_{1},u_{1}\right)\wedge p\left(r_{1},b_{1},v_{1}\right)\wedge J\left(u_{1},v_{1},x_{2}\right)>\theta . \end{array}$$

Thus *J*(*u*_1_, *v*_1_, *x*_2_) > *θ* and so *u*_1_ ⊕ *B* ~ *x*_2_ ⊕ *B*.

Since

(43) $$\begin{array}{c} \left(r_{2}⊙\left(a_{1}⊕b_{2}\right)\right)\left(x_{3}\right)>J\left(a_{1},b_{2},a_{3}\right)\wedge p\left(r_{2},a_{3},x_{3}\right)>\theta , \end{array}$$

we have ((*r*_2_⊙*a*_1_)⊕(*r*_2_⊙*b*_2_))(*x*_3_) > *θ*. So there exist *u*_2_ ∈ *A*, *v*_2_ ∈ *B* such that

(44) $$\begin{array}{c} p\left(r_{2},a_{1},u_{2}\right)\wedge p\left(r_{2},b_{2},v_{2}\right)\wedge J\left(u_{2},v_{2},x_{3}\right)>\theta . \end{array}$$

Similarly, we get *J*(*u*_2_, *v*_2_, *x*_3_) > *θ* and so *u*_2_ ⊕ *B* ~ *x*_3_ ⊕ *B*.

Since

(45) $$\begin{array}{c} \left(\left(r_{1}\circ r_{2}\right)⊙a_{1}\right)\left(x_{1}\right)>G\left(r_{1},r_{2},r\right)\wedge p\left(r,a_{1},x_{1}\right)>\theta , \end{array}$$

we get ((*r*_1_⊙*a*_1_)⊕(*r*_2_⊙*a*_1_))(*x*_1_) > *θ*. Hence

(46) $$\begin{array}{c} p\left(r_{1},a_{1},u_{1}\right)\wedge p\left(r_{2},a_{1},u_{2}\right)\wedge J\left(u_{1},u_{2},x_{1}\right)>\theta . \end{array}$$

Therefore, since *u*_1_ ⊕ *B* ~ *x*_2_ ⊕ *B*, *u*_2_ ⊕ *B* ~ *x*_3_ ⊕ *B*, *J*(*x*_2_, *x*_3_, *y*_1_) > *θ*, and *J*(*u*_1_, *u*_2_, *x*_1_) > *θ*, we obtain *x*_1_ ⊕ *B* ~ *y*_1_ ⊕ *B* and [*x* ⊕ *B*] = [*y* ⊕ *B*].

(3) Finally, let ((*r*_1_∗*r*_2_)  ⊙  [*a* ⊕ *B*])([*x* ⊕ *B*]) > *θ* and (*r*_1_  ⊙  (*r*_2_  ⊙  [*a*  ⊕ *B*]))([*y*  ⊕  *B*]) > *θ* and let *r* ∈ *R* such that *H*(*r*_1_, *r*_2_, *r*) > *θ*. Then there exist $a_{1},a_{2}\in \bar{a}$, $x_{1}\in \bar{x}$, $y_{1}\in \bar{y}$, *x*_2_, *x*_3_ ∈ *A*, and *b*_1_, *b*_2_ ∈ *B* such that

(47) $$\begin{array}{c} H\left(r_{1},r_{2},r\right)\wedge p\left(r,a_{1},x_{1}\right)>\theta ,\\ p\left(r_{2},a_{2},x_{2}\right)\wedge p\left(r_{1},x_{2},y_{1}\right)>\theta ,\\ J\left(a_{1},b_{1},a\right)>\theta ,\;\;J\left(a_{2},b_{2},a\right)>\theta , \end{array}$$

and let *w* ∈ *A* such that *p*(*r*, *a*_2_, *w*) > *θ*. By

(48) $$\begin{array}{c} \left(r_{1}⊙\left(r_{2}⊙a_{2}\right)\right)\left(y_{1}\right)>p\left(r_{2},a_{2},x_{2}\right)\wedge p\left(r_{1},x_{2},y_{1}\right)>\theta ,\\ \left(\left(r_{1}*r_{2}\right)⊙a_{2}\right)\left(w\right)>H\left(r_{1},r_{2},r\right)\wedge p\left(r,a_{2},w\right)>\theta , \end{array}$$

we get *w* = *y*_1_ and *p*(*r*, *a*_2_, *y*_1_) > *θ*.

Since *a*_1_ ⊕ *B* ~ *a*_2_ ⊕ *B*, *p*(*r*, *a*_1_, *x*_1_) > *θ*, and *p*(*r*, *a*_2_, *y*_1_) > *θ*, then we have *x*_1_ ⊕ *B* ~ *y*_1_ ⊕ *B* from Proposition 28 and consequently [*x* ⊕ *B*] = [*y* ⊕ *B*].

**Theorem 30 (fundamental homomorphism theorem of fuzzy modules)**

Let (*R*, *G*, *H*) be fuzzy ring and *f* : *A* → *B* an *R*-fmodule epimorphism. Then *A*/*K* is isomorphic to *B* where *K* = Ker⁡  *f*.

**Proof**

We have shown that *K* = Ker⁡  *f* is a fuzzy submodule of *A* in Proposition 26. Hence *A*/*K* is an *R*-fmodule from Theorem 29. Now we define a mapping by

(49) $$\begin{array}{c} \overset{-}{f}:\frac{A}{K}⟶B,\\ \left[a⊕K\right]⟼f\left(a\right). \end{array}$$

$\overset{-}{f}$ is well-defined, one to one and surjective fuzzy group homomorphism from Theorem  5.3 in [7]. Then it suffices to prove that, for any *r* ∈ *R*, *a* ∈ *A*,  $\bar{p}(r[a⊕K],[b⊕K])>\theta$ implies $\bar{p}(r,\overset{-}{f}([a⊕K]),\overset{-}{f}([b⊕K]))>\theta$. Let $\bar{p}(r[a⊕K],[b⊕K])>\theta$ and let *u* ∈ *B* such that $\bar{p}(r,\overset{-}{f}([a⊕K]),u)>\theta$. Then we have $a^{\prime }\in \bar{a},b^{\prime }\in \bar{b}$ such that *p*(*r*, *a*′, *b*′) > *θ*.

Let *w* ∈ *A* such that *p*(*r*, *a*, *w*) > *θ*. Since *f* is an *R*-fmodule homomorphism, we get *p*(*r*, *f*(*a*), *f*(*w*)) > *θ*, so *u* = *f*(*w*).   By *a*′  ⊕  *K* ~ *a*  ⊕  *K*, *p*(*r*, *a*′, *b*′) > *θ*, *p*(*r*, *a*, *w*) > *θ*, and Proposition 28, we have *b*′ ⊕ *K* ~ *w* ⊕ *K* and so [*b* ⊕ *K*] = [*w* ⊕ *K*]. Therefore $f(w)=\;\overset{-}{f}([w⊕K])=\overset{-}{f}([b⊕K])=f(b)$ and consequently *u* = *f*(*b*).
